# Correcting for non-compliance when determining colonic transit time with radio-opaque markers

**DOI:** 10.3748/wjg.v23.i4.740

**Published:** 2017-01-28

**Authors:** Alvin Ibarra, Kaisa Olli, Arthur C Ouwehand

**Affiliations:** Alvin Ibarra, Kaisa Olli, Arthur C Ouwehand, DuPont Nutrition and Health, 02460 Kantvik, Finland

**Keywords:** Colonic transit time, Gastroenterology, Gut transit time, Radio-opaque marker, X-ray

## Abstract

The use of radio-opaque markers and abdominal X-ray is the standard method for determining colonic transit time (CTT). However, when there are deviations in the intake of these markers by participants in clinical trials it is desirable to improve observations by introducing corrections, where possible. To date, there is no standard procedure to adjust for such deviations. This report proposes a series of alternatives based on possible scenarios for deviations from the intended intake of radio-opaque markers. The proposed method to correct for missed or delayed consumption of radio-opaque markers can help to increase the accuracy of the CTT measurements in clinical trials.

**Core tip:** Ibarra A et al correcting for non-compliance when determining colonic transit time with radio-opaque markers.

## TO THE EDITOR

The use of radio-opaque markers and abdominal X-ray is the standard approach for determining colonic transit time (CTT)[1,2]. This technique is simple, inexpensive, reliable and reproducible[1,2]. The principle of the method is based on the consumption of radio-opaque markers for six consecutive days, creating an equilibrium between incoming and outgoing markers, followed by an abdominal X-ray on day 7. The number of markers that can be identified on the X-ray is an expression of CTT per the following equation:

CTT = *n_i_* × (*t/N*)

where *n_i_* is the number of markers that is observed on X-ray, *t* is the time between the ingestion of markers in hours and *N* is the total number of markers that is ingested each day. Thus, if markers are consumed at 24-h intervals and the number of markers per day is 24, CTT equals the total marker count on the X-ray[1,2].

This method was developed to diagnose constipation subtypes: those with “normal” transit times and those with “slow transit”, the latter of which can be subdivided into “colonic inertia”, “hindgut dysfunction” and “outlet obstruction”[1,2]. In addition, this protocol is used for research purposes to study the effects of certain dietary interventions on CTT.

This procedure requires compliance with marker consumption and abdominal X-ray. However, patients and volunteers in nutritional studies might fail to adhere to the protocol. Non-compliance can lead to substantial underestimation of CTT. Bouchoucha and colleagues[3] determined the influence of non-compliance on the diagnosis (delayed transit and site of the delay) and concluded that skipping the ingestion of markers for one or two days still allows for an acceptable clinical diagnosis.

However, it is unknown whether non-compliance is also tolerable for dietary intervention studies. Dietary intervention studies that involve, for example, fiber, prebiotics and probiotics will often aim to detect modest changes in CTT[4]. Non-compliance with radio-opaque marker consumption is likely to increase the variations between baseline and treatment values and between those of the treatments and placebo, impeding the ability to detect subtle changes due to a nutritional intervention. Thus, what may be acceptable for a diagnosis might be unsuitable for dietary research. In this report, we propose a method for correcting some of the errors that are introduced by non-compliance with radio-opaque marker consumption and X-ray.

Table 1 lists various scenarios for radio-opaque marker consumption. In these examples, it is assumed that 24 markers are to be consumed per day. Scenario 1 represents full compliance with marker consumption; thus, as discussed above, the number of markers that are observed indicates the CTT. Deviations from the marker consumption protocol can be divided in two types: those that have occurred “outside” of versus “within” the calculated CTT. To examine the influence of non-compliance and determine whether a correction can be performed to mitigate it, the day and time at which the non-compliance occurred must be known. Instances of non-compliance that took place “outside” of the CTT can be assumed to have had no influence on the transit time as calculated (Scenario 2). Had the markers that were omitted or consumed at the wrong time been ingested as the protocol requires, they could reasonably be assumed to have been excreted (Scenario 3). Thus, Scenarios 2 and 3 do not require any correction and are assumed to have accurately estimated the CTT, consistent with Bouchoucha and colleagues[3].

**Table 1:** Examples of scenarios of compliance and non-compliance with radio-opaque marker consumption and their potential correction

**Days**	**Scenario 1**	**Scenario 2**	**Scenario 3**	**Scenario 4**	**Scenario 5**	**Scenario 6**	**Scenario 7**
1	●	●	●	●	●	●	●
2	●	●	●	●	●	●	●
3	●	● (± α h)	○	●	●^1^	●	●
4	●^1^	●^1^	●	●^1^	●^1^	○	● (+ 4 h)^1^
5	●^1^	●^1^	●	● (± β h)^1^	●^1^	●^1^	●^1^
6	●^1^	●^1^	●^1^	●^1^	○^1^	●^1^	●^1^
*n*	24	24	24	24	24	24	72
*t* (h)	24	24	24	24	24	24	72 (-4)
*ni* (X-ray)	70	52	24	60	69 (+24)	48	65
CTT (h)	70	52	24	60	93	48	61.4

1 The period indicated by the estimated colonic transit time (CTT)-*i.e*., the markers on the days that are visualized on the X-ray. Scenarios 1 to 6: *n* is the total number of markers ingested each day, *t* is the time between marker ingestion in hours, *ni* is the number of markers observed on X-ray, and CTT is the estimated colonic transit time. Scenario 7: *n* is the total number of markers ingested during the estimated CTT period, *t* is the number of days in hours on which the markers are detected minus the number of hours by which consumption is delayed, *ni* is the number of markers observed on X-ray, and CTT is the estimated colonic transit time. Missed markers are indicated as open circles, consumed markers are indicated as closed circles. α and β are hypothetical deviation times of radio-opaque marker consumption. Values between parenthesis indicate corrections.

When a day on which the markers were consumed too early or too late falls “within” the calculated CTT (Scenario 4), no correction is needed, because all markers will be observed, regardless of whether they are consumed at the scheduled time. If the markers are missed (*i.e*., not taken) on a day “within” the calculated CTT, we can assume that had those markers been consumed, they would have been detected by X-ray (Scenario 5). Thus, a correction can be made, adding the missed markers. A similar correction can be made if the X-ray is delayed by a day (*i.e*., without providing extra radio-opaque markers), because such a case can be treated as if the markers that were scheduled for the last day were not consumed (Scenario 5). When a marker has been missed just outside of the calculated CTT (Scenario 6), no correction can be made, because it is uncertain whether the markers, if consumed, would have been detected. When radio-opaque markers have been consumed too late and when these markers are the “oldest” that are still observed, according to the calculated CTT (Scenario 7), a correction can be applied, assuming that instead of *t* = 24 (in the example), *t* is the number of days (in hours) on which the markers are detected minus the number of hours by which consumption was delayed, and *N* is the number of markers that was consumed during this period.

To correct the various scenarios accurately, the exact day and time of non-compliance of radio-opaque marker intake during the intervention must be known. These values can be tracked with a diary that is completed by the subject throughout the administration of radio-opaque markers according to the protocol, helping to identify the precise time of non-compliance.

In conclusion, the proposed method to correct for missed or delayed consumption of radio-opaque markers can help to increase the accuracy of the CTT measurements in clinical trials.

## References

[1] Bouchoucha M, Devroede G, Dorval E, Faye A, Arhan P, Arsac M (2006). Different segmental transit times in patients with irritable bowel syndrome and “normal” colonic transit time: is there a correlation with symptoms?. Tech Coloproctol.

[2] Bouchoucha M, Devroede G, Faye A, Le Toumelin P, Arhan P, Arsac M (2006). Colonic response to food in constipation. Int J Colorectal Dis.

[3] Bouchoucha M, Prado J, Chtourou L, Devroede G, Atanassiu C, Benamouzig R (2011). Non-compliance does not impair qualitative evaluation of colonic transit time. Neurogastroenterol Motil.

[4] Miller LE, Ouwehand AC (2013). Probiotic supplementation decreases intestinal transit time: meta-analysis of randomized controlled trials. World J Gastroenterol.

